# Do All Roads Lead to Rome? Genes Causing Dravet Syndrome and Dravet Syndrome-Like Phenotypes

**DOI:** 10.3389/fneur.2022.832380

**Published:** 2022-03-11

**Authors:** Jiangwei Ding, Lei Wang, Zhe Jin, Yuanyuan Qiang, Wenchao Li, Yangyang Wang, Changliang Zhu, Shucai Jiang, Lifei Xiao, Xiaoyan Hao, Xulei Hu, Xinxiao Li, Feng Wang, Tao Sun

**Affiliations:** ^1^Ningxia Key Laboratory of Cerebrocranial Disease, The Incubation Base of National Key Laboratory, Ningxia Medical University, Yinchuan, China; ^2^Department of Neurosurgery, General Hospital of Ningxia Medical University, Yinchuan, China; ^3^Department of Neurosurgery, The First Affiliated Hospital of Xinxiang Medical University, Weihui, China; ^4^Department of Medical Cell Biology, Uppsala University, Uppsala, Sweden; ^5^Department of Neurology, First Affiliated Hospital of Zhengzhou Universiy, Zhengzhou, China; ^6^Department of Neurosurgery, The Fifth Affiliated Hospital of Zhengzhou University, Zhengzhou, China; ^7^Department of Neurosurgery, The First Affiliated Hospital, Zhejiang University School of Medicine, Hangzhou, China

**Keywords:** Dravet syndrome, severe myoclonic epilepsy in infancy, *SCN1A*, *PCDH19*, epilepsy gene

## Abstract

**Background:**

Dravet syndrome (DS) is a severe epileptic encephalopathy mainly caused by haploinsufficiency of the gene *SCN1A*, which encodes the voltage-gated sodium channel Na_V_1. 1 in the brain. While *SCN1A* mutations are known to be the primary cause of DS, other genes that may cause DS are poorly understood. Several genes with pathogenic mutations result in DS or DS-like phenotypes, which may require different drug treatment approaches. Therefore, it is urgent for clinicians, especially epilepsy specialists to fully understand these genes involved in DS in addition to *SCN1A*. Particularly for healthcare providers, a deep understanding of these pathogenic genes is useful in properly selecting and adjusting drugs in a more effective and timely manner.

**Objective:**

The purpose of this study was to identify genes other than *SCN1A* that may also cause DS or DS-like phenotypes.

**Methods:**

A comprehensive search of relevant Dravet syndrome and severe myoclonic epilepsy in infancy was performed in PubMed, until December 1, 2021. Two independent authors performed the screening for potentially eligible studies. Disagreements were decided by a third, more professional researcher or by all three. The results reported by each study were narratively summarized.

**Results:**

A PubMed search yielded 5,064 items, and other sources search 12 records. A total of 29 studies published between 2009 and 2021 met the inclusion criteria. Regarding the included articles, seven studies on *PCDH19*, three on *SCN2A*, two on *SCN8A*, five on *SCN1B*, two on *GABRA1*, three on *GABRB3*, three on *GABRG2*, and three on *STXBP1* were included. Only one study was recorded for *CHD2, CPLX1, HCN1* and *KCNA2*, respectively. It is worth noting that a few articles reported on more than one epilepsy gene.

**Conclusion:**

DS is not only identified in variants of *SCN1A*, but other genes such as *PCDH19, SCN2A, SCN8A, SCN1B, GABRA1, GABRB3, GABRG2, KCNA2, CHD2, CPLX1, HCN1A, STXBP1* can also be involved in DS or DS-like phenotypes. As genetic testing becomes more widely available, more genes associated with DS and DS-like phenotypes may be identified and gene-based diagnosis of subtypes of phenotypes in this spectrum may improve the management of these diseases in the future.

## Introduction

Dravet syndrome (DS), also known as severe myoclonic epilepsy in infancy (SMEI), is a catastrophic developmental and epileptic encephalopathy with onset in infancy that was initially described and reported by Dravet in 1978 (1) to distinguish it from Lennox–Gastaut syndrome (LGS). As increasing reports found that patients did not present with myoclonic seizures and that seizures appeared not to be limited to infancy and childhood but persisted through adulthood (2), the International League Against Epilepsy named DS as a distinct syndrome in 1989 (3). The estimated incidence of DS ranges from 1 in 20,000 to 1 in 40,000 (4).

DS is often associated with developmental delays, severe cognitive deficits, sleep disorders, behavioral disorders, including autistic-like behavior, and increased risk of sudden unexpected death in epilepsy (SUDEP; nearly 20%) (5–7). DS is a severe developmental and epileptic encephalopathy typically caused by loss-of-function de novo mutations in the *SCN1A* gene that encodes the voltage-gated sodium channel isoform Na_V_1.1 (8–10).

The voltage-gated sodium channel comprises a primary α subunit (composed of six transmembrane domains, Na_V_1.1–Na_V_1.9, encoded by the genes *SCN1A–SCN11A*) and a secondary β subunit (a single transmembrane domain, β1–β4, encoded by the *SCN1B–SCN4B* genes (11). The α subunit forms the sodium channel pore and is the target of various antiepileptic drugs. The β subunits interact with α subunit and modulate the localization of alpha subunits and channel properties (10, 12).

Na_V_1.1 encoded by *SCN1A* is a membrane protein of 2,009 amino acids formed by four domains each containing six transmembrane segments. In the central nervous system, Na_V_1.1 is mainly expressed in cell bodies and dendrites, but also at the axon initial segments of some interneurons, which play a very important role in the generation and propagation of action potential (13–15). Mutations in the *SCN1A* gene lead to a significant decrease in sodium current in GABAergic interneurons, which influences GABA inhibitory function and leads to neuronal hyperexcitability and seizures (16, 17). In addition, reduced sodium current can also affect Purkinje cells, resulting in motor dysfunction, and can lead to behavioral problems and cognitive dysfunction (16, 18).

*SCN1A* mutation is the most common pathogenic factor of DS, and about 80% of DS patients have *SCN1A* mutations (19, 20). More than 1,800 pathogenic *SCN1A* variants have been identified to date (21). Most variants are de novo, but there are individuals who carry the *SCNA1* mutation in one or both parents <10%; (19, 22). Patients with *SCN1A* mutations have varying manifestations, ranging from mild and drug-responsive epilepsy, such as genetic epilepsy with febrile seizures plus (GEFS+), to developmental and epileptic encephalopathy, including DS, West syndrome, and myoclonic-atonic epilepsy (23–25). Since about 80% of DS is caused by *SCN1A* mutations (20, 26), clinicians have sufficient knowledge of the association between *SCN1A* and DS, but DS caused by genes other than *SCN1A* are insufficiently understood and little is known about them (26). Herein, we review and discuss the latest evidence on other genes that are potentially involved in DS or DS-like phenotypes.

## Methods

**Figure 1:**
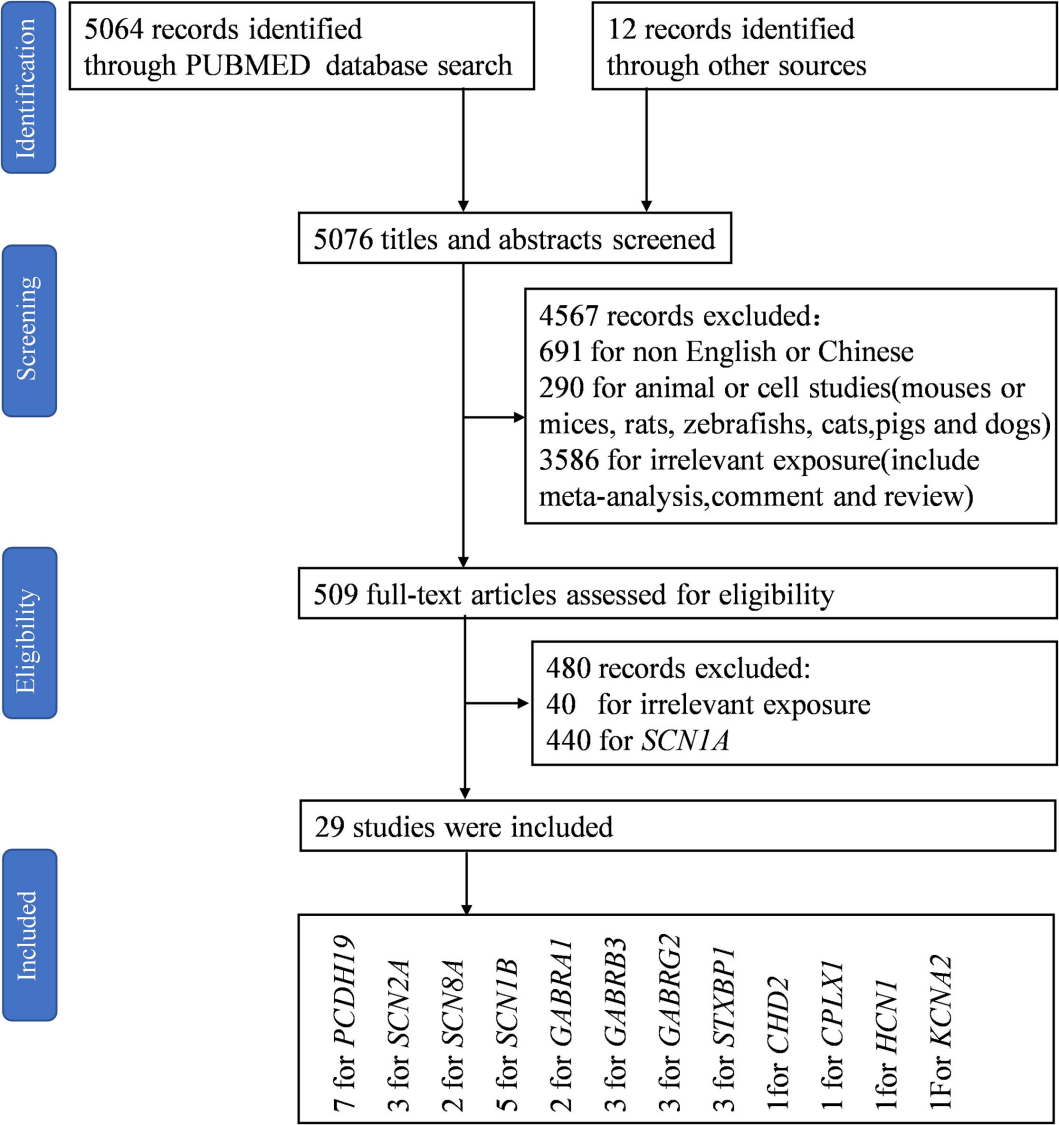
Flow diagram of included studies. “P*CDH19, SCN8A*, and *STXBP1*” and “*GABRA1* and *STXBP1*” appeared in the same study, respectively (27, 28). Liu et al. reported the same epilepsy gene in two papers (29, 30).

Using PubMed, we conducted a systematic literature search of studies published up to December 1, 2021, selecting research that examined genes other than *SCN1A* that are associated with DS. The search terms were selected from the thesaurus of the National Library of Medicine (Medical Subject Heading Terms, MeSH) and included the terms “Dravet syndrome” and “severe myoclonic epilepsy in infancy.” The final search equation was defined using the Boolean connectors “OR” following the formulation “Dravet syndrome” OR “severe myoclonic epilepsy in infancy.” The search included English or Chinese-language articles published from November 11, 1947, to November 27, 2021, and did not include any subheadings or tags (i.e., search fields “All fields”; Figure 1).

### Inclusion and Exclusion Criteria

Our objective was to investigate the infrequent or rare pathogenic genes of DS; thus, all genes associated with DS except *SCN1A* met the inclusion criteria. We excluded articles not written in English or Chinese, non-original work that was unrelated to human subjects, such as reviews, meta-analyses, animal or cell subjects, and experimental articles without information related to the question posed in this review. Records were screened by Jiangwei Ding and evaluated by Lei Wang with respect to the inclusion and exclusion criteria. Discrepancies were discussed and decided by Tao Sun or all three.

## Results

### Study Selection

Of 5,076 references identified in the initial search in the electronic databases, the study selection process resulted in the inclusion of a total of 29 studies in the narrative synthesis (Figure 1).

### Study Characteristics and Findings

The study characteristics included in this review are shown in Table 1, Figure 2. All studies involved DS or SMEI and were published from 2009 to 2021 in English or Chinese. Regarding the studies included, six studies on *PCDH19* (27, 29–34), three on *SCN2A* (36–38), two on *SCN8A* (27, 39), five on *SCN1B* (40–44), two on *GABRA1* (28, 45), three on *GABRB3* (47–49), three on *GABRG2* (50–52), and three on *STXBP1* (27, 28, 46) were included. Only one study was recorded for *CHD2* (54), *CPLX1, HCN1*, and *KCNA2*, respectively (35, 53, 55, 56). We mapped the available mutations onto the protein topologies (protein topologies from Protter - interactive protein feature visualization (ethz.ch).

**Table 1:** The data of DS-like caused by multiple single-gene mutations that met the inclusion criteria were summarized.

**Study**	**Mutation gene**	**Total case**	**DS-like case**	**Mutation site**
Depienne et al. (31)	*PCDH19*	73	10 (9 female +1 male)	c.142G>T/p. Glu48X
				c.352G>T: p. Glu118X
				c.859G>T:/p. Glu287X
				c.3319 C>G/p. Arg1107Gly
				c.506delC/p.Thr169Serfs ×43
				c.1036_1040dup/p. Asn347Lysfs ×23
				c. 361G>A/p. Asp121Asn
				c.595 G>C/p. Glu199Gln
				c.1019A>G/p. Asn340Ser
				c.1628 T>C/p. Leu543Pro
Kwong et al. (32)	*PCDH19*	18	1 (female)	p.D377N
Liu et al. (29) and Liu et al. (30)	*PCDH19*	75	6 (female)	c.488T>G/ p. V163G
				c.1347-1348insAAC/p. N449-H450insN
				c.1019A>G/ p. N340S
				c.1019A>G/ p. N340S
				c.695G>A/ p. N232S
				c.1091dupC/p. Y366Lfs ×10
Higurashi et al. (33)	*PCDH19*	116	7 (female)	c.1019-1092insC/p.Try336Leufs**×**10
				c.840 C>G/ p. Tyr280X
				c.215 T>G/ p. Val72Gly
				c.571G>C/ p. Val191Leu
				c.949C>T/ p. Gln317X
				c.1019A> G/ p. Asn340Ser
				c.772_773del AT/p.Ile258Profs ×61
Trivisano et al. (34)	*PCDH19*	15	15	Not available
Liu et al. (35)	*PCDH19*	36	5	Not available
Shi et al. (36)	*SCN2A*	59	1	c.3935G>C/p. R1312T
Zeng et al. (37)	*SCN2A*	21	1	c.4988T>C/p. I1663T
Lossin et al. (38)	*SCN2A*	1	1	p. R1312T
Larsen et al. (39)	*SCN8A*	17	3	g.52200671C>G/ p. Gln1801Glu
				g.52200885G>A/p. Arg1872Gln
				g.52093426T>C/p. Phe260Ser
Liu et al. (35)	*SCN8A*	36	1	Not available
Patino et al. (40)	*SCN1B*	1	1	c.373C>T/p. R125C
Ogiwara et al. (41)	*SCN1B*	68	1	c.316A>T/p. Arg125Cys
Mukherjee et al. (42)	*SCN1B*	1	1	chr19:35524568; C>T/p. R125C
Gong et al. (43)	*SCN1B*	22	2	c.351C>T/p. G117G
				c.467C>T/ p. T156M
Darras et al. (44)	*SCN1B*	2	2	c.265C>T/ p.Arg89Cys
Johannesen et al. (45)	*GABRA1*	26	9	c.226A>C/p. S76R
				c.335G>A/p. R112Q
				c.335G>A/ p. R112Q
				c.335G>A/p. R112Q
				c.436C>A/p. L146M
				c.641G>A/p. R214H
				c.751G>A/p. G251S
				c.875C>T/p. T292I
Carvill et al. (28)	*GABRA1*	13	4	c.751G>A/p. Gly251Ser
				c.335G>A/Arg112Gln
				c.335G>A/Arg112Gln
				c.917A>C/Lys306Thr
	*STXBP1*		3	c.847G>A/Glu283Lys
				c.853G>T/Asp285Tyr
				c.1334A>C/His445Pro
Álvarez Bravo et al. (46)	*STXBP1*	1	1	p. Arg406His
Liu et al. (35)	*STXBP1*	36	1	Not available
Le et al. (47)	*GABRB3*	6	1	c.695G>A/ p. Arg232Gln
Shi et al. (48)	*GABRB3*	1	1	p. R232Q
Pavone et al. (49)	*GABRB3*	1	1	c.842 C>T/p. Thr281IIe
Huang et al. (50)	*GABRG2*	1	1	p. Q40X
Ishii et al. (51)	*GABRG2*	2	2	c.118C>T/p. Q40X
Hernandez et al. (52)	*GABRG2*	1	1	p. P302L
Syrbe et al. (53)	*KCNA2*	33	6	p. P405L
				p. P405L
				p. P405L
				p. I263T
				p. R297Q
				p. L298F
Suls et al. (54)	*CHD2*	9	3	c.18102A>C (p.?)
				c.4971G>A/p. Trp1657*
				c.1396C>T/p. Arg466*
Redler et al. (55)	*CPLX1*	3	3	c.315C4A/p. Cys105Ter
				c.315C4A/p. Cys105Ter
				c.382C4A/p. Leu128Met
Nava et al. (56)	*HCN1^#^*	157	6	c.140G>T/p. Gly47Val
				c.299C>T/p. Ser100Phe
				c.814T>C/p. Ser272Pro
				c.835C>T/p. His279Tyr
				c.890G>C/p. Arg297Thr
				c.1201G>Cp.Asp401His

# *These patients were initially suggestive of DS but showed different progression over time*.

**Figure 2:**
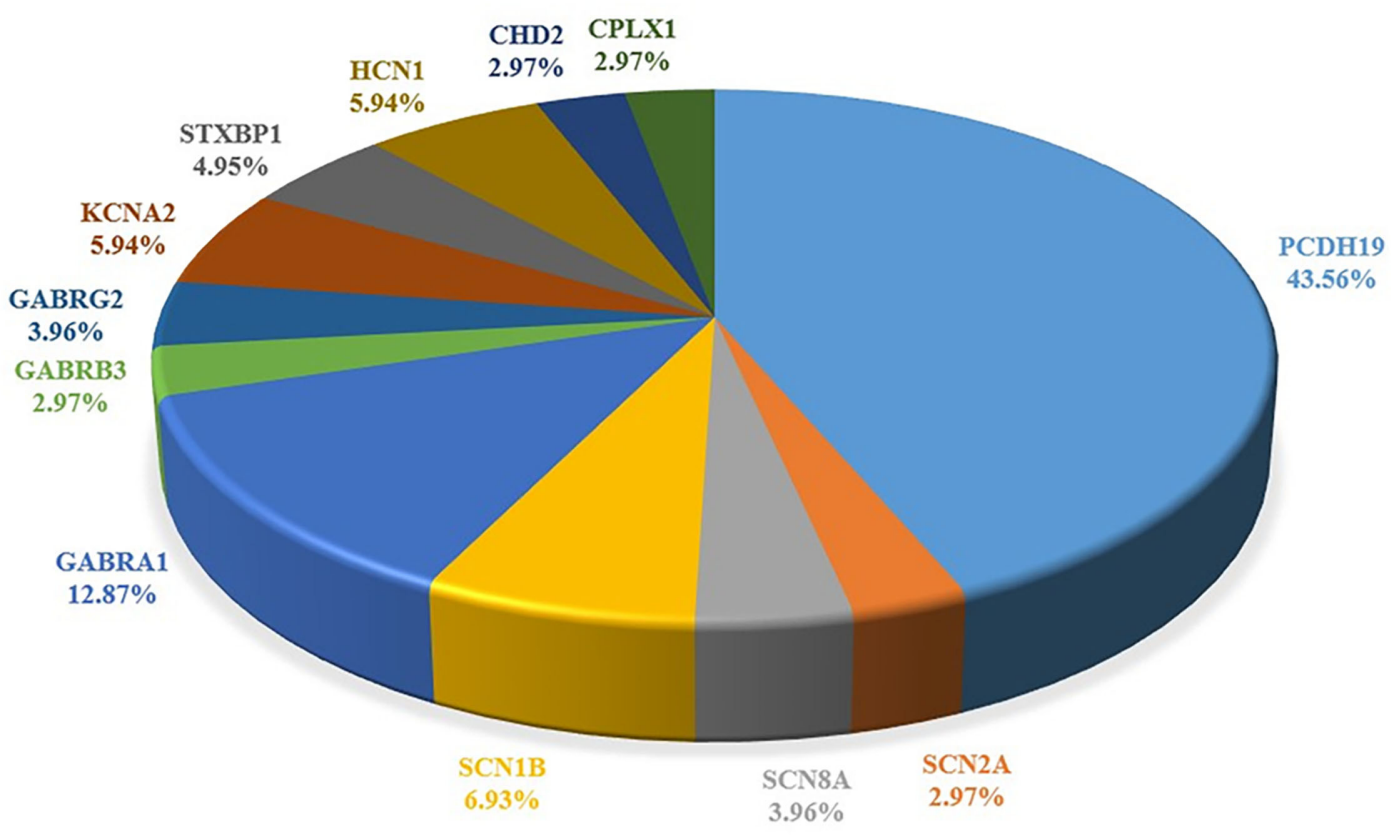
Summary of the pathogenic genes of Dravet syndrome. Comparison of proportion of each gene in literature reports.

## Discussion and Synthesis

DS is an early infantile epileptic and developmental encephalopathy and is mostly caused by loss-of-function mutations in the brain sodium channel Na_V_1.1 (6). *SCN1A* variants are known to cause a variety of other diseases, including epilepsy (Doose syndrome, epilepsy of infancy with migrating focal seizures, west syndrome, LGS, and Rett syndrome) and non-epileptic disorders (familial/sporadic hemiplegic migraines, autism spectrum disorder, sudden infant death syndrome, and arthrogryposis multiplex congenita) in addition to DS (24, 57–60). Conversely, could DS be caused by mutations in a variety of genes other than *SCN1A*? The answer seems to be clear from the data in the articles included in our study, which indicate that at least 13 genes, including *SCN1A*, have been identified as directly involved in DS to date.

According to our data, in addition to *SCN1A*, there are 12 genes that can cause DS or DS-Like phenotypes, among which *PCDH19* is the most common, making it the second-most common pathogenic gene of DS. The other genes were three sodium channel–related genes (*SCN2A, SCN8A*, and *SCN1B*), one potassium ion channel–related gene (*KCNA2*), three gamma-aminobutyric acid receptors (GABAR) genes (*GABRA2, GABRB3*, and *GABRG2*), one cyclic nucleotide gated cation channel gene (*HCN1*), and other functional genes including *CHD2, CPLX1*, and *STXBP1* (Table 1; Figure 2).

### PCDH19

**Figure 3:**
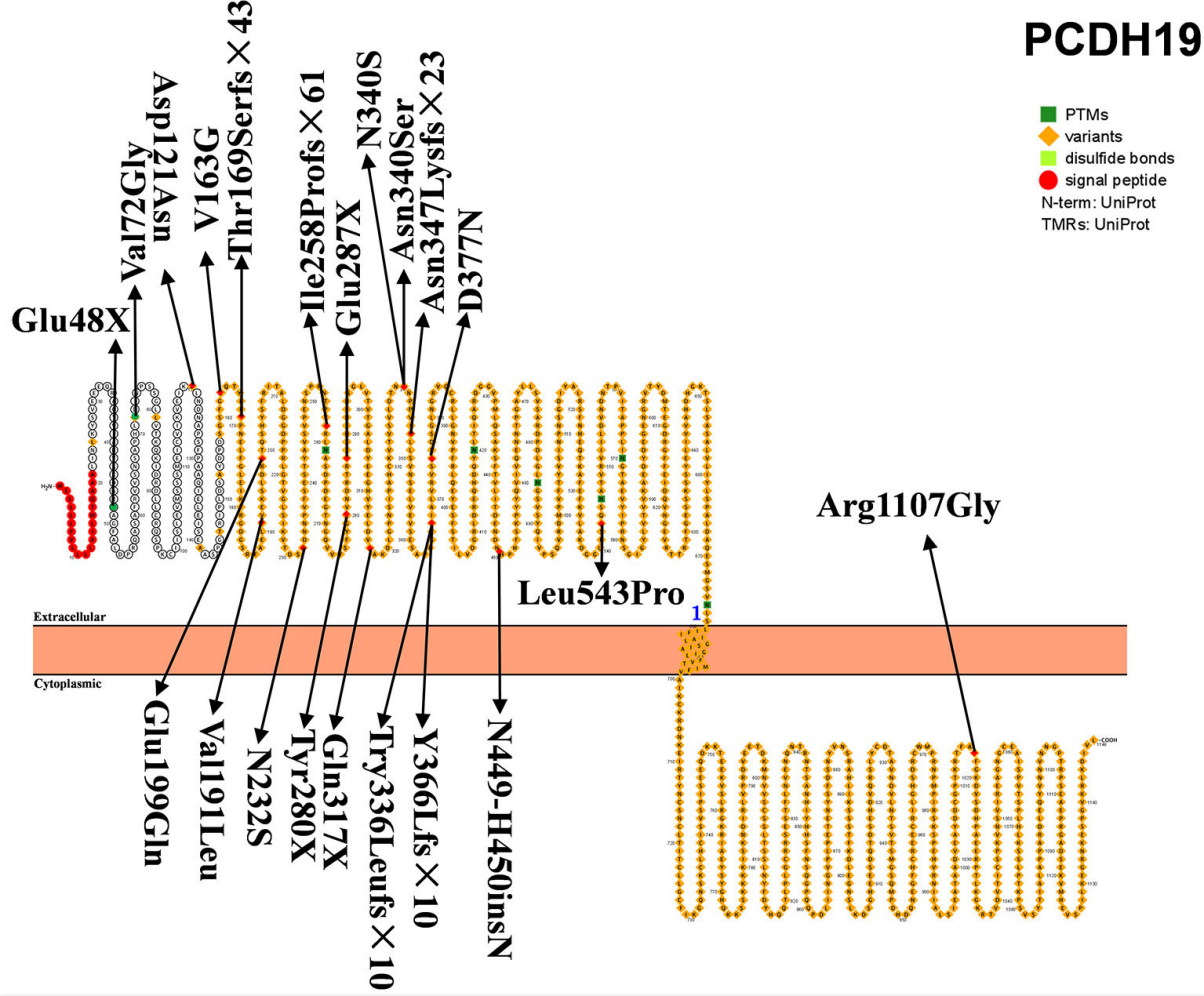
Schematic diagram of protein encoded by *PCDH19*, illustrating the location of the amino acids affected by the mutations identified in patients.

*PCDH19*, located on chromosome XQ22.1, encodes the protocadherin-19 and is the second-most pathogenic gene of DS, accounting for 23.4% of patients with *SCN1A*-negative DS (61). The product of *PCDH19* is protocadherin-19, a single-channel transmembrane glycoprotein, which is the largest subgroup of the cadherin superfamily, and plays an important role in cell adhesion, dendritic self-avoidance, and axon guidance. *PCDH19* consists of six exons and encodes 1,148 amino acids (Figure 3). This protein includes one signal peptide, six extracellular cadherin repeats, one transmembrane region, and one cytoplasmic region. Exon 1 encodes more than half of the proteins, including all extracellular and transmembrane domains. *PCDH19* is highly conserved in humans, mice, zebrafish, and chickens, and is highly expressed in central nervous system tissues at different developmental stages, including the subventricular area, intermediate area, inferior plate, specific cerebral cortex, hippocampus, and cerebellum. It has been speculated that *PCDH19* is involved in the establishment of neuronal connections and the signal transmission of synaptic membranes (62). *PCDH19* gene mutation was first reported by Dibbens et al. (63) in seven families with epilepsy and intellectual disability confined to women. *PCDH19-related DS* main clinical feature is epilepsy with intellectual disability and onset in infancy or childhood. It is a special X-linked hereditary epilepsy, and the heterozygous female family members carrying the *PCDH19* mutation are affected. Hemizygous men with the mutation are not affected. However, it has been subsequently reported that men with chimeric mutations of *PCDH19* also develop the disease (31).

Depienne et al. found the mutation of *PCDH19* in 15% of *SCN1A*-negative DS patients. This was the first time the relationship between *PCDH19* and DS was described and analyzed, indicating that *PCDH19* may be a DS or DS-like pathogenic gene (31). Subsequently, a large number of reports and descriptions of *PCDH19* mutation causing DS were published (33, 34). A recent review showed that the incidence of DS with *SCN1A* mutation was significantly higher than that of DS patients with *PCHDH19* mutation (76.6 vs. 23.4%), while the incidence of autism was higher in DS patients with *PCDH1*9 variants [62.5 vs. 37.5%, respectively; (61)]. The patients with *PCDH19* variance had obvious tendencies toward hyper-febrile seizures. Patients with *PCDH19* and *SCN1A* mutations had very similar clinical characteristics, including the association of early febrile and non-febrile seizures, cluster seizures, developmental and language delays, behavioral disorders, and cognitive degradation (32). However, *PCDH19* mutations rarely reveal status epilepticus, photosensitive seizures, clonic seizures, myoclonic seizures, and absence seizures. Furthermore, the seizures gradually decrease after school age, and the prognosis is generally better than that of DS. *PCDH19* is currently considered to be the second-most common pathogenic gene for DS (but much less common than *SCN1A*).

### SCN2A

**Figure 4:**
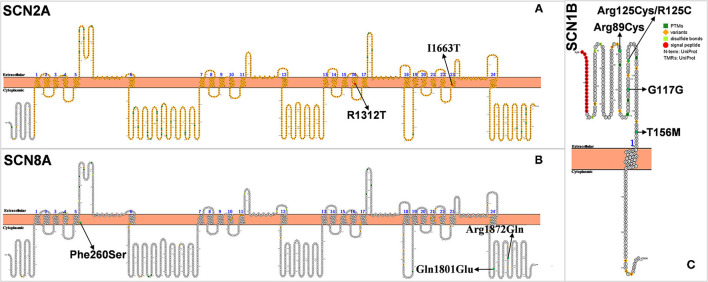
**(A–C)** Schematic diagram of protein encoded by *SCN2A, SCN8A*, and *SCN1B*, illustrating the location of the amino acids affected by the mutations identified in patients.

The *SCN2A* gene, encoding voltage-gated sodium channel α subunit Na_V_1.2, is located on chromosome 2q24.3 and contains 26 exons and encodes 2,005 amino acids (Figure 4A). Na_V_1.2 is widely distributed in the brain and highly expressed in the cortex, hippocampus, striatum, and midbrain (64). *SCN2A* is the main pathogenic gene of benign familial neonatal–infantile epilepsy (BFNIE) (65). *SCN2A* mutations can lead to a wide clinical spectrum of epilepsy, such as benign familial neonatal epilepsy (BFNE), benign familial infantile epilepsy (BFIE), and developmental epileptic encephalopathy such as DS, Ohtahara syndrome, and epilepsy of infancy with migrating focal seizures (EIMFS), West syndrome, Doose Syndrome, LGS, and unclassified epileptic encephalopathy (38, 66, 67).

In 2009, Japanese scholars Shi et al. found for the first time three *SCN2A* missense mutations in 59 patients with DS, one of which was a *de novo SCN2A* mutation (36). In 2012, Lossin et al. also found that *SCN2A* mutation (c.3935G>C/p. R1312T) could cause DS. This mutation is thought to affect the position of arginine at the channel voltage sensor, which in turn causes conformational changes in the ion channel and opens the otherwise closed channel pore, which may be responsible for the pathogenesis of DS (38). Chinese scholars Zeng et al. retrospectively analyzed 21 patients with *SCN2A* mutation and found that one of them was diagnosed with DS (37). The gene mutation in this patient was inherited from his mother. The onset of the disease was at 6 months of life. The patient mainly had generalized tonic-clonic seizures (GTCS), myoclonic seizures (MS), and febrile seizures (FS). His symptoms did not improve at the 42-month follow-up, and his intellectual and motor development was delayed. These studies suggest that *SCN2A* is one of the epileptogenic genes in DS.

### SCN8A

*SCN8A*, encoding voltage-gated sodium channel Na_V_1.6, is located on chromosome 12q13.13 (Figure 4B) (68). Na_V_1.6 is the main voltage-gated sodium channel in the mammalian central nervous system. It is highly concentrated at the axon hillock and plays an important role in depolarization during the generation of neuronal action potential (69). Sodium-channel sequences are highly conserved during evolution, and even small changes in biophysical properties can have significant effects *in vivo* (70). In 2012, Veeramah et al. first discovered and reported the mutation of *SCN8A*(c.5302A>G/p.Asn1768Asp), which is also the fifth sodium-channel gene involved in epilepsy (68). More than 140 cases of *SCN8A* mutation-associated epilepsy have been reported in children (71).

Within *SCN8A*-related encephalopathy, individuals have been diagnosed with syndromes including unclassified EE (epileptic encephalopathy), early infantile EE, LGS, West syndrome, and DS-like. Similar to DS, children with *SCN8A* epilepsy also present with speech problems and intellectual retardation/developmental delay (72). In 2015, Larsen et al. found three DS-like patients with *SCN8A* mutation (39). The core symptoms of the *SCN8A*-related DS-like phenotype are markedly different from those of the typical Dravet syndrome caused by the *SCN1A* mutation. The average age of onset of DS-like caused by *SCN8A* mutation in the three patients was 4.6 months (3, 7, and 4 months, respectively), earlier than that of the typical DS. Only one patient had fever-induced seizures and a few patients had convulsive seizures. In contrast, typical DS often starts with fever-induced seizures and rarely presents with convulsive seizures. The development of the patient is normal before the onset of seizures, but when seizures start occurring, mental, motor, and language skills show gradual retardation, and autistic symptoms similar to those of DS. Recently, Liu et al. found an *SCN8A* mutation in 36 DS patients but did not describe the symptoms (27).

DS is caused by a dominant loss-of-function mutation in *SCN1A* that leads to reduced Na_V_1.1 activity, resulting in insufficient excitability of GABAergic neurons, while the gain of function mutations in *SCN8A* can lead to severe epileptic encephalopathy subtypes through overactivation of the Na_V_1.6 channel and of glutamatergic neurons (73). These observations suggest that Na_V_1.1 and Na_V_1.6 represent two opposing aspects of the neural balance between inhibition and activation. This difference may be responsible for the effect of sodium channel blockers such as oxcarbazepine and lamotrigine on *SCN8A*-related epilepsy while exacerbating *SCN1A*-related epilepsy.

*SCN8A* can also be considered as a modifier gene of *SCN1A*-induced DS. To test this hypothesis, Martin and his colleagues generated *Scn1a* (+/-); *Scn8a*(med-jo/+) in double-heterozygous mice. They found that *Scn1a* (+/-); *Scn8a*(med-jo/+) mice had a similar susceptibility to epilepsy as wild-type mice, unlike *Scn1a* (+/-) mice. The *Scn8a*(med-jo) allele was also able to rescue the premature lethality of *Scn1a*(+/-) mice and extended the lifespan of *Scn1a*(-/-) mutants (74). Lenk et al. (75) recently used antisense oligonucleotide (ASO) to reduce the transcription of *Scn8a* in mice with epileptic encephalopathy and *SCN1A-*related DS and found a 25–50% reduction in delayed seizure onset and lethality, which was consistent with the results of Martin et al. (74, 75). It was further confirmed that *SCN8A* is not only an epilepsy gene, but also a modification gene of epilepsy.

### SCN1B

*SCN1B*, encoding the voltage-gated sodium channel (VGSC) β1 non–pore-forming subunits, is the first gene confirmed to be associated with GEFS+ pathogenesis located on 19q13.11 (76, 77) (Figure 4C). The mutation of *SCN1B* plays an important role in the pathogenesis of febrile seizures plus spectrum diseases and is associated with epilepsy syndromes including febrile seizures (FS), GEFS+ (77, 78). The reported mutations of epileptogenic *SCN1B* are all missense mutations except for one splice-site mutation. Heterozygous missense mutations mostly cause FS or GEFS+ with mild symptoms, while homozygous missense mutations lead to DS severe symptoms (40–43).

The relationship between *SCN1B* and DS has been the focus since Patino et al. reported that the first patient with *SCN1B* mutant showed DS-like symptoms (40). They reported a case of homozygous *SCN1B* mutation in an infant with heat-dependent seizures triggered by vaccination and fever, consistent with some of the characteristics of DS. However, the age of onset at 3 months, abnormal electrical discharge on EEG, psychomotor deterioration at 5 months, and eventual death from respiratory pneumonia at about 14 months all preceded and were more severe than DS (40). Mukherjee et al. also reported on a patient with an *SCN1B* mutation (42). The patient had an onset of GTCS from 3 months of age after vaccination. Seizures then recurred at 4 months of age due to fever, by which time a global developmental delay had occurred. The age of onset of epilepsy and cognitive dysfunction in this patient were prematurely earlier than that of DS. According to the diagnosis of DS, the onset is about 6–12 months, and cognitive functioning may deteriorate until 1–2 years old. However, the authors suggest that, as the child was having post-vaccinal convulsions, a possible diagnosis of DS was considered, which is clearly inappropriate (42). Aeby et al. recently reported a patient with *SCN1B* mutation who was hypotonic at birth and presented with multiple seizures (multifocal myoclonus, focal seizures, and myoclonic status epilepticus) at 2.5–3 months of age, associated with fever. The patient's auditory brainstem response (ABR) showed bilateral hearing loss. After taking fenfluramine, the frequency of seizures was significantly reduced, and status epilepticus disappeared after 2 years of follow-up. The authors diagnosed it as early infantile developmental and epileptic encephalopathy (DEE) rather than DS (79).

Nevertheless, some cases with *SCN1B* mutations that could be considered as DS have been reported. Ogiwara et al. found one case with a homozygous *SCN1B* mutation in 67 patients with DS but without *SCN1A* and *SCN2A* mutations (41). Gong et al. also discovered two *SCN1B* mutations in 22 DS patients without *SCN1A* mutations (43). Recently, Darras et al. identified two DS patients with *SCN1B* mutations. However, these two patients had mild symptoms that differed markedly from the previous cases reported by Patino and Mukherjee et al. (40, 42). O'Malley et al. reported that adult *Scn1b* knockout mice showed DS-like phenotype such as epilepsy and SUDEP (80). In summary, *SCN1B* mutation is not a common cause of DS-like, if any (81).

### KCNA2

**Figure 5:**
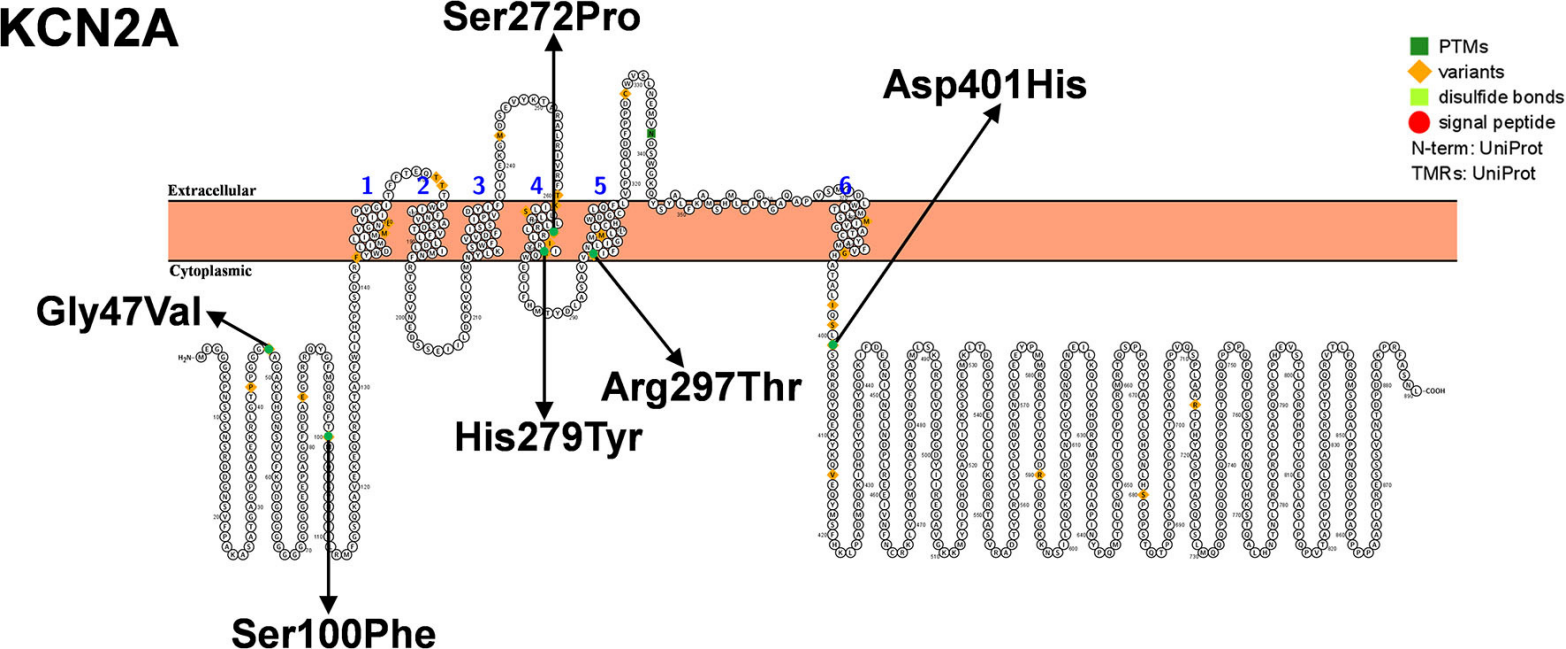
Schematic diagram of protein encoded by *KCN2A*, illustrating the location of the amino acids affected by the mutations identified in patients.

The *KCNA2* gene encoding the K_v_1.2 channel is located on chromosome 1p13.3, with a total length of about 3,100 bp, containing three exons and two introns, and encoding 499 amino acids (Figure 5). The K_v_1.2 channel consists of four subunits with six transmembrane (S1–S6). The transmembrane segments S1–S4 form a voltage sensor domain, and S5–S6 form a pore region containing selective filters and gate ion flow (82, 83). K_v_1.2 is mainly expressed in the axons and presynaptic terminals of neurons (84). In 2015, Pena and Coimbra (85) performed exome analysis in a 7-year-old boy who presented with ataxia and myoclonic epilepsy as an infant and identified a novel *KCNA2* mutation (c.890C>A/p. Arg297Gln). In the same year, Syrbe et al. identified four different *de novo* variants in six patients (one mutation recurred three times independently) with epileptic encephalopathy, including one with clinical symptoms similar to DS (53). In animal model studies, *Kcna*2 knockout mice were found to be more prone to epilepsy and premature death than wild-type mice, which further confirms the important role of *KCNA2* in the pathogenesis of epilepsy including DS (86).

### GABRA1

**Figure 6:**
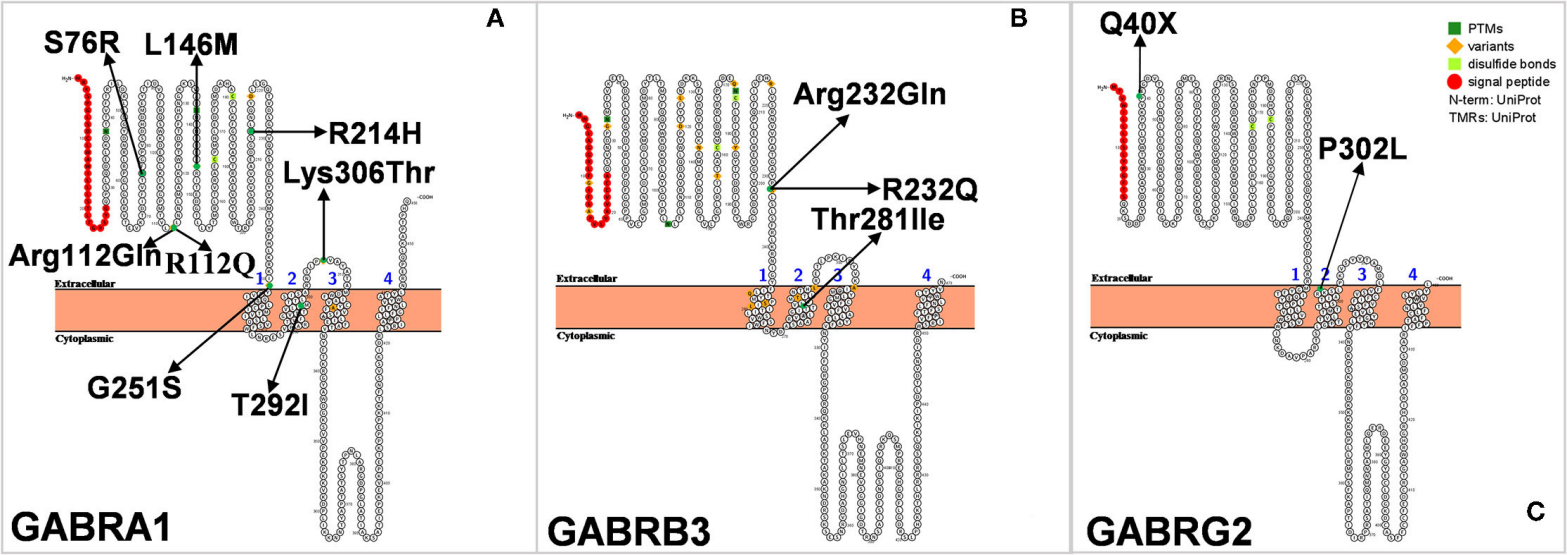
**(A–C)** Schematic diagram of protein encoded by *GABRA1***, ***GABRB3*, and *GABRG2*, illustrating the location of the amino acids affected by the mutations identified in patients.

Gamma-aminobutyric acid receptors type A (GABAAR) are ligand-gated chloride ion channels that mediate synaptic inhibition in the brain. GABAAR is a hetero-pentameric protein complex composed of different subunits (α1-6, β1-3, γ1- 3, δ, ε, π, θ, and ρ1-3) (87, 88). The combination α1β2γ2 is thought to be the most abundant form of receptor in the brain. In 2002, Cossette et al. (89) first discovered a mutation of the *GABRA1* encoding the α1 subunit (Ala322Asp) in an idiopathic generalized epilepsy (IGE) patient (Figure 6A). Furthermore, they constructed cells carrying Ala322Asp mutation and found that cells expressing the mutant GABAAR (α1Ala322Aspβ2γ2) had a lower amplitude of GABA-activated currents than those expressing the wild-type (α1β2γ2) receptor. Similarly, LaChance-Touchette et al. also found *GABRA1* mutations in IGE (90). In a recent study, a *GABRA1*-deficient zebrafish model exhibited reduced activity and defective expression of other GABAAR subunits (91). In addition to IGE, *GABRA1* mutations are also closely associated with Ohtahara syndrome and West syndrome, suggesting that *GABRA1* is not only involved in mild genetic generalized epilepsies and febrile seizures, but is also associated with early-onset epileptic encephalopathies [EOEEs; (28, 92)].

*GABRA1* mutation is also rare in Dravet syndrome. In 2014, Carvill et al. sequenced the genes of seven patients with *SCN1A*-negative DS and found that four of them were associated with *GABRA1* mutation. The four patients had an average age of onset of epilepsy at 9 months of age, and all their seizures were associated with fever. Two of the patients had developmental delays, which might have contributed to the diagnosis of DS, but their full clinical details have not been published (28). In 2016, Johannesen et al. evaluated 26 patients with epilepsy caused by *GABRA1* mutation, of whom five were diagnosed with DS and four were diagnosed with DS-like. Febrile seizures are the first symptom in most patients diagnosed with DS, whereas tonic-clonic and hemiclonic seizures are the main symptoms of DS-like disorders. The age of onset of DS was later than that of DS-like disorder (9.5 months vs. 6.3 months) (45). All of the above studies directly verified the correlation between *GABRA1* and DS or DS-like.

### GABRB3

*GABRB3* is located in the 15q11.2-q12 region and encodes theβ3 subunit of the GABAAR (Figure 6B). A large number of reports have indicated that the variation of *GABRB3* may be related to the occurrence of childhood absence epilepsy (CAE) (93). In addition, the reduced expression of GABRB3 is also closely associated with Rett syndrome, Angelman syndrome, and autism spectrum disorders (94). The common characteristics of these diseases are epilepsy and neurodevelopmental delay. DS is a refractory epileptic encephalopathy, accompanied by febrile seizures, spontaneous epilepsy, intellectual disability, autism, and other complications. Could mutations in *GABRB3* also cause DS? In 2017, Le et al. carried out exome sequencing on six patients with *SCN1A*-negative DS and found a new heterozygous missense variant of *GABRB3*, and thus *GABRB3* was considered to be a candidate gene for DS (47). Recently, Piero et al. also found a novel *GABRB3* missense mutation in an 18-year-old girl with DS (49). The patient experienced two febrile seizures when she was 7 months old. Electroencephalography revealed bilateral slow waves mainly in the occipital regions. The seizures were poorly controlled with oral antiepileptic drugs, and progressive rapid motor and cognitive impairments appeared after several months. While growing up, her social skills were still poor, and her intelligence quotient (IQ) was low (IQ = 42). These prompted the authors to make a diagnosis of DS. Unlike patients with typical DS, the patient was found to have dysmorphism of the spine with dorsal hyperkyphosis at the age of 15 months.

### GABRG2

The *GABRG2* gene, located on chromosome 5, encodes the γ2 subunit of GABAAR (Figure 6C), which plays an important role in receptor transport to the membrane and aggregation in the postsynaptic membrane. In 2002, Kananura et al. first found the splicing donor mutation of the *GABRG2* gene (IVS6+2T → G) in a patient with childhood absence epilepsy and febrile seizures as the main clinical manifestations (95). We have previously found that mice selectively knocked out of *Gabrg2* had temperature-dependent epilepsy (96). Up to now, about 20 mutations of *GABRG2* have been reported, including Q40X, Q390X, R136X, W429X, Q40X, R136X, Q390X, and W429X. Mice carrying the *GABRG2* mutation (P.R82Q) showed less cortical inhibition, spontaneous spiny discharges, and heat-induced seizures (97). *GABRG2* mutations result in variable clinical phenotypes, ranging from mild symptoms for FS, CAE, and rolandic epilepsy (RE) to severe GEFS+ to epileptic encephalopathies (EEs) such as DS, West syndrome, and Doose syndrome, which may differ due to mutations at different sites. To date, only four cases of *GABRG2* mutation causing DS have been reported, including a pair of twins (50–52). This is the only case occurring in twins that was reported in the literature with a simple symptom description. The patients came from a Japanese twin family and had experienced seizures since they were 2 months old. One of the twins died at 3 years old, and the other died at 5 months old. Interestingly, their father, who also had the same *GABRG2* mutation, had no seizure episodes, while their mother, who did not carry the mutation, had several seizure episodes during childhood (51).

### CHD2

**Figure 7:**
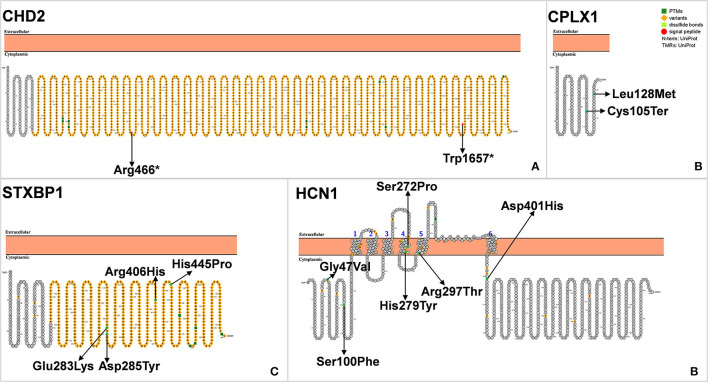
**(A–D)** Schematic diagram of protein encoded by *CHD2, CPLX1, STXBP1*, and *HCN1A*, illustrating the location of the amino acids affected by the mutations identified in patients.

Chromodomain helicase DNA-binding (CHD) protein belongs to the SNF2-related ATPase superfamily, which uses the energy of ATP hydrolysis to change the position of nucleosomes and reorganize chromatin structures. CHD2 belongs to a subfamily of the CHD protein family, which plays a unique role in human brain development and function. The *CHD2* gene is located on 15q26, with a total length of 128 951 bp, and its protein coding region contains 39 exons (Figure 7A) (98, 99). In 2009 Veredice et al. reported the first *CHD2* mutation in a 30-month-old female infant with myoclonic encephalopathy (100). She began to have seizures at 6 months of age, presenting as distinct generalized clonic seizures of approximately 35 min duration, followed by seizures several times a day; seizures were also observed to be sensitive to intermittent light stimulation, which could be reduced by wearing sunglasses. In addition to epilepsy, she also showed mild intellectual disability [developmental quotient (DQ) = 67]. Carvill et al. conducted massive parallel resequencing of 19 known and 46 candidate genes in 500 patients with epileptic encephalopathy, among which six *CHD2* mutations were found (101).

Although *CHD2* is associated with epilepsy, there have been few reports of *CHD2* mutations in DS (54).

Unlike *SCN1A* mutation-associated DS, the onset of seizures in these patients was at more than 1 year of age (mean age: 26.6 months), and the seizures were heat-sensitive and accompanied by mild cognitive impairment. Some patients also show symptoms of ataxia and dysarthria (54). Suls et al. performed whole-exome sequencing on genomic DNA of the nine selected individuals with *SCN1A*-negative DS and both unaffected parents and found three *CHD2* mutations. They then knocked down *Chd2* in zebrafish using targeted morpholine antisense oligomers. Epileptiform discharges were recorded and confirmed on the *Chd2*-knockdown larvae, which were similar to seizures in affected patients (54). Therefore, *CHD2* gene testing is recommended for DS with negative *SCN1A* gene mutation and accompanied by myoclonic seizures and intellectual disability. Current genetic testing methods (parallel sequencing) will significantly increase the probability of *CHD2* mutant detection.

### CPLX1

*CPLX1* encodes complexin 1, a presynaptic small molecule protein that belongs to the highly conserved complexin protein family. *CPLX1* forms a soluble N-ethylmaleimide–sensitive factor-attachment protein receptor (SNARE) complex in the central nervous system involved in the anchoring, pre-excitation, and fusion of axonal end vesicles (102, 103). The abnormal expression of *CPLX1* is seen in several neurodegenerative and psychiatric disorders including schizophrenia, Alzheimer's disease, Huntington's disease, major depressive illness and bipolar disorder (103–105). Studies using knockout rodent models have shown that *CPLX1* is associated in neurological and psychiatric disorders, such as social disorder ataxia and premature death (106, 107). Although *CPLX1* is associated with a variety of neurological disorders, little is known about its association with epilepsy. In 2015, Karaca and colleagues found *CPLX1* gene mutation in two patients with malignant epilepsy (108). In 2017, Redler et al. identified three *CPLX1* mutations in patients with SMEI, which was the first reported association between SMEI and *CPLX1* (Figure 7B) (55). Distinctly different from typical DS, SMEI caused by the *CPLX1* mutation can result in non-febrile seizures, with an alarming number of seizures up to several hundred times per day. The most characteristic *CPLX1* mutations are associated with mild craniofacial dysmorphisms.

### STXBP1

Syntaxin-binding protein 1 (STXBP1), also known as Munc18-1 or SM protein, is a membrane transporter primarily expressed in the brain. This protein plays an important role in synaptic signal transduction by interacting SNARE to mediate the release of synaptic vesicles (109). *STXBP1* is located on chromosome 9q34.11 and contains 20 exons. STXBP1 is composed of 594 amino acids and contains three domains, namely domains 1, 2 and 3, which form a bow structure (Figure 7C).

New heterozygous mutations of *STXBP1* can cause early-onset epileptic encephalopathy and neurodevelopmental disorders. The *STXBP1* mutation causes a wide range of febrile epileptic syndromes, ranging from simple febrile seizures to severe epileptic encephalopathy (110). In 2008, Saitsu et al. first identified four *STXBP1* heterozygous missense mutations in four patients with Otahara syndrome (three males and one female) and identified *STXBP1*as the pathogenic gene (111). In seven *SCN1A*-negative DS patients, Carvill et al. found three patients with *STXBP1* mutation, of which one patient presented with onset at >1 year old; the two other patients presented with both tonic and non-tonic seizures. All three patients presented with febrile seizures with severe cognitive impairment, which could not be distinguished from those in typical DS based on the published clinical data (28). Alvarez Bravo et al. performed genetic testing on a 19-year-old female patient with *SCN1A-*positive DS and also found that the patient had *STXBP1* mutation. At 6 months old, the patient had a generalized seizure due to infection and fever. Cognitive retardation began at 2 years of age and became more severe at 6 years of age. She had a myoclonus triggered by sound and tactile stimuli at the age of 13 years. At the age of 12 years, she had a severe movement disorder. Unlike typical DS, this patient had the movement disorder Parkinson's disease (112). Patients with *STXBP1* mutation have DS-like phenotypes regardless of *SCN1A* mutations, and *STXBP1* has been included as a rare candidate gene for DS (28).

### HCN1

Hyperpolarization-activated cyclic nucleotide-gated channel 1 (*HCN1*) plays a very important role in regulating the resting membrane potential of neurons and transmitting synaptic information (113). Nava et al. found two *HCN1* mutations in two proband. Three *HCN1* mutants were found to be in a heterozygous state in 95 febrile French early infantile epileptic encephalopathy (EIEE) cohort patients. A novel c.835C>T (p. His279Tyr) mutation was found in a Dutch follow-up cohort of 62 patients. For all six patients with *HCN1* mutation in EIEE, the initial suggested diagnosis was DS (Figure 7D) (56). It is worth noting that the patients showed different clinical manifestations that deviated from DS over time. These patients may present with features consistent with some fragments of DS in the early stages of the disease (114).

## Conclusion

DS is not only identified in variations of *SCN1A*; mutations in other genes such as *PCDH19, SCN2A, SCN8A, SCN1B, KCNA2, GABRA1, GABRB3, GABRG2, CHD2, CPLX1, HCN1A*, and *STXBP1* are rare but can also occur in DS-like phenotypes. Although *SCN1A*-related-Dravet syndrome currently accounts for most DS diagnoses and the relationship between *SCN1A* mutations and DS is highly specific (21), the diagnosis and treatment of other gene-related-Dravet-like syndromes should not be ignored. It is important to highlight that some phenotypes initially considered as DS have now been reclassified as DS-like ones in the spectrum of developmental and epileptic encephalopathies (21). As genetic testing becomes more widely available, more genes associated with DS or DS-like may be identified and gene-based diagnosis of different subtypes may improve the management of these diseases in the future.

## Data Availability Statement

The original contributions presented in the study are included in the article/supplementary material, further inquiries can be directed to the corresponding authors.

## Author Contributions

All authors listed have made a substantial, direct, and intellectual contribution to the work and approved it for publication.

## Funding

This work was funded by National Natural Science Foundation of China, Grant/Award Numbers: 81971085 and 81860220; the Ningxia Hui Autonomous Region 13th Five-Year Plan Major Science and Technology Projects (2002170101); and the Advantages Discipline Group Project of Ningxia Medical University, Grant/Award Number: XY201511.

## Conflict of Interest

The authors declare that the research was conducted in the absence of any commercial or financial relationships that could be construed as a potential conflict of interest.

## Publisher's Note

All claims expressed in this article are solely those of the authors and do not necessarily represent those of their affiliated organizations, or those of the publisher, the editors and the reviewers. Any product that may be evaluated in this article, or claim that may be made by its manufacturer, is not guaranteed or endorsed by the publisher.

## Abbreviations

DS: Dravet syndrome
EE: epileptic encephalopathy
LGS: lennox-gastaut syndrome
SMEI: severe myoclonic epilepsy in infancy.
